# Analyzing the influence of mobile internet on urban rail transit travel behavior in Chongqing, China: Social informatization as mediating variables

**DOI:** 10.1371/journal.pone.0331977

**Published:** 2025-11-20

**Authors:** Yuyi Zhu

**Affiliations:** School of Architecture and Urban Planning, Chongqing University, Chongqing, China; Southwest Jiaotong University, CHINA

## Abstract

The rapid development of mobile internet technology and the widespread adoption of smartphones have significantly influenced people’s daily lives and complicated the mechanisms affecting urban rail transit travel behavior. While existing studies focus predominantly on developed countries, research remains limited in megacities in developing nations experiencing rapid technological growth. Using questionnaire data from urban rail transit travelers in Chongqing, China, this study investigates the intrinsic relationship between mobile internet and residents’ urban rail transit travel behavior, along with its underlying mechanisms. Key findings reveal that: 1) Transportation infrastructure and management informatization directly influence residents’ urban rail transit travel behavior; 2) Transportation services informatization exerts indirect effects; 3) Information literacy and resources mediate the influence of transportation informatization on urban rail transit travel behavior. These findings not only enhance the understanding of how mobile internet is transforming urban rail transit travel behavior but also provide a theoretical foundation for optimizing urban spatial organization in the future.

## 1. Introduction

With the iterative advancement of mobile internet technology and the widespread adoption of smartphone applications, the information exchange methods within human society have undergone fundamental changes [1]. This transformation has further redefined urban transportation systems; traditional continuous and fixed travel modes have gradually been disrupted, giving rise to a new type of urban transportation characterized by fragmented temporal and spatial distribution and flexible travel options [2,3]. According to QuestMobile data, as of June 2024, China had 1.235 billion monthly active mobile internet users, with megacities significantly surpassing the national average. This trend suggests a strong correlation between mobile internet applications and urban mobility patterns [4].

As the primary mode of transportation in high-density urban areas, urban rail transit has evolved from a singular means of transport into a complex system that integrates infrastructure, public space, and data nodes [5,6]. Despite extensive literature demonstrating that mobile internet positively influences urban rail transit travel behavior [7], two notable limitations persist: First, regarding mechanism analysis, most existing studies focus on examining direct impacts and lack systematic verification of the action paths of mediating variables, such as residents’ information literacy and the availability of information resources. Second, the geographical representativeness of previous research is insufficient, with empirical studies in this field largely concentrated in developed countries, such as Japan [8], Canada [9], and Singapore [10]. These findings may not be applicable to developing countries, as they overlook the comprehensive impact of rapid technological advancements and infrastructure expansion on urban areas in these regions. Although mobile internet technology initially spread from urban areas in developed countries, its influence has widely permeated most cities in developing countries. This trend underscores the necessity of focusing on developing countries, as they may provide a more representative average. Additionally, it is essential to concentrate on representative megacities, as they may better reflect the typical urban experience.

Therefore, analyzing the impact of mobile internet on urban rail transit travel behavior in cities within developing countries, particularly in representative megacities, is essential for understanding the developmental trends of urban transportation systems. This analysis will enhance the existing research and provide theoretical support for the sustainable development of urban areas.

In the context of developing countries, mobile internet technology is increasingly integrated into residents’ daily travel behaviors, significantly influencing the decision-making processes and behavioral patterns associated with rail transit travel. However, there is currently a lack of systematic understanding regarding the specific mechanisms of this influence, particularly concerning the multidimensional responses of travel behavior, the intrinsic pathways of technological intervention, and their mediating mechanisms. Therefore, this study focuses on the following core questions: How does mobile internet technology affect residents’ urban rail transit travel behavior? What structure does the intrinsic relationship of this influence exhibit? How are the pathways of its effects generated and altered? To address these questions, this research focuses on the urban rail transit travel behavior of residents in Chongqing, aiming to explore the mechanisms by which mobile internet technology intervenes in urban rail transit travel behavior and to assess its potential for optimizing travel experiences and enhancing the efficiency of multifaceted elements in rail transit systems. The specific research objectives include: 1) systematically identifying the multidimensional factors influencing urban rail transit travel behavior due to mobile internet technology, clarifying its intervention mechanisms in areas such as travel cognition, route selection, and time management; 2) constructing a dynamic pathway model of technological influence on urban rail transit travel behavior to elucidate the logic of behavioral changes under technological impact; 3) examining the mediating variables in the relationship between technological intervention and behavioral responses, while quantifying their regulatory effects. The academic value of this research lies in enriching the theoretical framework of urban rail transit travel behavior and its integration with information technology, thereby providing scientific theoretical support for optimizing residents’ rail transit travel experiences in the information age.

## 2. Literature review

### 2.1. Mobile internet and urban transport informatization

The urban transportation system is a vital component of contemporary urban development, encompassing various elements such as transportation participants (travelers, drivers, and managers), goods, vehicles, and the transportation infrastructure [11]. The integration of mobile internet technologies has significantly enhanced the interconnectivity of these elements by providing multiple means of information delivery, including voice, text, images, and video [12]. Despite the considerable potential of mobile internet to improve efficiency and modernize the management of transportation operations [13,14], existing research has not yet fully explored how mobile internet specifically enhances the level of service of transportation infrastructure and how these improvements contribute to the sustainability of urban transportation systems. These issues represent key areas that require in-depth analysis in future research.

### 2.2. Factors influencing urban rail transit travel behavior

Urban rail transit travel behavior is a complex decision-making process influenced by multiple factors. Prior research has identified key individual attributes that affect travel behavior, including age, education level, travel experience, and travel preferences [15]. Additionally, personal psychological factors, such as considerations of safety, privacy, and social needs, can significantly impact travel decisions [16,17]. Empirical studies have demonstrated that years of mobile internet use and the number of hours spent on personal smartphones are positively associated with the frequency of informational travel [18]. Highly educated individuals tend to have more experience with information-based travel, which explains why travelers who are active on mobile applications are primarily characterized by a young, well-educated, and technologically savvy demographic [19].

Residents’ urban rail transit travel behavior is influenced by both the transportation and travel environments. On one hand, the transportation environment encompasses the infrastructure and essential services that facilitate travel for residents. This includes the layout of the transportation network, the variety of available transportation modes, the accessibility of transportation facilities, and the reliability of transportation services, all of which significantly impact travel behavior. In particular, the connectivity and coverage of the transportation network play a crucial role in determining residents’ choice of mode [20]. On the other hand, the social environment, which includes travel culture, values, and technological advancements, also subtly influences residents’ travel behavior [21,22].

Existing studies have confirmed the positive impact of mobile internet on the development of urban transportation systems; however, the specific effects, nature, and pathways through which mobile internet influences urban rail transit travel behavior have yet to be explored in depth. As a diverse and complex system, the potential impacts and mechanisms of mobile internet on travel behavior at various levels warrant further investigation.

## 3. Methods

To reveal the primary factors influencing mobile internet usage on urban rail transit travel behavior, this study explores the relationship pathways between the two and identifies key mediating variables along with their mechanisms. The research methods are closely aligned with these objectives: First, the study selected 30 urban rail transit stations in the main urban area of Chongqing to conduct a questionnaire survey, using systematic sampling to collect data on urban rail transit travel behavior and basic personal information. Second, through a literature review, we identified three potential variables: transportation informatization, social informatization, and residents’ urban rail transit travel behavior, along with relevant explanatory factors. A 17-item, 5-point Likert scale was developed, and four sets of research hypotheses were proposed, that is, H1 (transportation informatization directly affects urban rail transit travel behavior), H2 (transportation informatization promotes social informatization), H3 (social informatization affects travel behavior), and H4 (social informatization serves as mediating variables). Subsequently, SPSS 25.0 was utilized to test the reliability and validity of the data, while AMOS 25.0 was employed to construct a structural equation model. The Bollen-Stine bootstrap method was applied to optimize model fit, assess the direct and indirect effects between variables, and verify the impact of variables and the mediating role of social informatization in the relationship between mobile internet and urban rail transit travel behavior. Finally, through parameter and path analysis within the structural equation model, the key pathways and the strength of the mediating variables were quantified, providing empirical evidence for understanding the complex mechanisms of urban transit travel behavior in the context of informatization.

### 3.1. Study area

This paper focuses on Chongqing as a subject due to three significant aspects of its representation. First, from a spatial perspective, Chongqing, recognized as the world’s largest mountain city, features a unique three-dimensional transportation network and a high reliance on mobile internet technology. Second, in terms of technological advancement, as of 2023, Chongqing leads the western region in 5G infrastructure and smartphone penetration, boasting a penetration rate of 133.98 units per 100 people [23], which surpasses the national average of 122.5 units per 100 people [24]. Third, regarding transportation, Chongqing has developed the world’s largest mountainous urban rail transit network, with an operational length of 538 kilometers and a peak daily passenger flow of 5.081 million passengers. The average daily passenger flow and network coverage rank among the highest in China. Therefore, studying the role and impact of mobile internet technology in megacity urban rail transit travel in Chongqing is not only regionally representative but also holds significant theoretical and practical implications.

### 3.2. Data

**Fig 1:**
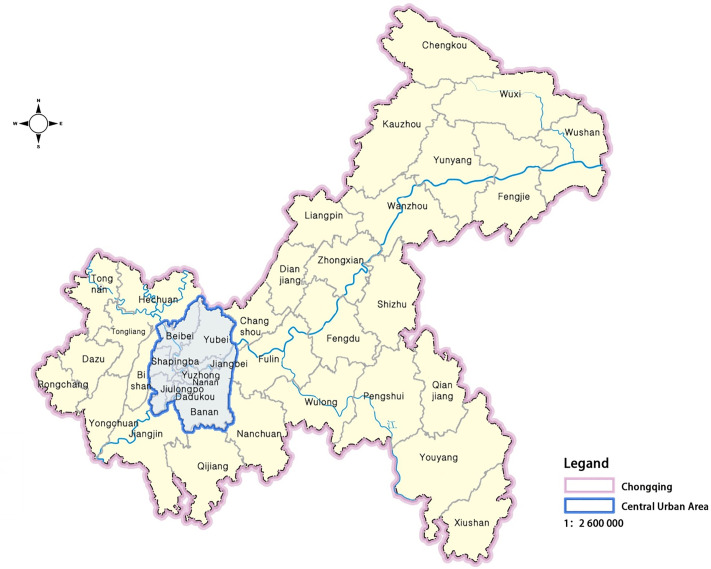
Study area. **Source:** http://bzdt.ch.mnr.gov.cn/browse.html?picId=%224o28b0625501ad13015501ad2bfc0230%22. (The image is sourced from the standard map service system website of the Ministry of Natural Resources of the People’s Republic of China. According to the website, standard maps may be used for news publications, book and newspaper illustrations, advertising displays, and craft design. The review number for this map is GS (2019) 3333).

The central urban areas of Chongqing were selected for the survey (Fig 1). Based on the passenger flow distribution characteristics of Chongqing’s rail transit stations, the 322 stations in the main urban area were categorized into four functional types: transportation hubs (13%), commercial services (19.3%), public services (19.3%), and residential areas (48.4%). To ensure the representativeness of the samples, 30 rail transit stations were randomly selected, including 4 transportation hubs, 6 commercial service stations, 6 public service stations, and 14 residential sites. From March 1 to June 1, 2024, paper-based questionnaires were distributed evenly during weekday peak hours (7:00–9:00, 17:00–19:00), off-peak hours (14:00–16:00), and weekend daytime (10:00–12:00), at the selected stations. Using systematic sampling, one passenger was selected for every five individuals to minimize interviewer bias. The survey targeted travelers; the questionnaire consisted of two parts: Part A gathered essential personal information about the travelers, which served as control variables for assessing the quality of the questionnaire, while Part B collected data on urban rail transit travel behavior. The control variables were adapted from a questionnaire developed by Bradley W. Lane [25], with appropriate modifications. The research project received a written exemption from the ethical review by the Ethics Committee of the School of Architecture and Urban Planning of Chongqing University.

Through the questionnaire survey, a total of 720 questionnaires were collected, of which 651 were deemed valid for further analysis, resulting in a response rate of 90.42%. The sample size (N = 651) is consistent with similar studies, such as Javid et al. (2021), which evaluated the attitudes of travelers in Lahore toward mobile applications with a sample size of N = 440 [26], and Saeipour et al. (2024), who collected data from 600 pedestrians in Urmia, Iran, using the “Pedestrian Behavior Questionnaire” to identify traffic behavior patterns [27]. In this survey, the respondents were primarily young and middle-aged adults. More than half (51%) held a bachelor’s degree or higher; 29.5% had 3–5 years of mobile internet experience, and 59.5% had more than 5 years (Table 1).

**Table 1:** The socioeconomic attributes of samples(N = 651).

Characteristics		Number	Percentage
Gender	Male	381	58.5%
	Female	270	41.5%
Age	19-30	274	42.1%
	31-50	321	49.3%
	Over 51	56	8.6%
Education level	Junior high school or less	153	23.5%
	High school or Technical secondary school	166	25.5%
	Junior college	102	15.7%
	Undergraduate	221	33.9%
	Graduate or higher	9	1.4%
Mobile internet experience(years)	1-2	72	11.1%
	3-5	192	29.5%
	6-9	201	30.9%
	Over 10	186	28.6%

### 3.3. Hypotheses and variables

This study employs structural equation modeling (SEM) to investigate how mobile internet usage influences urban rail transit travel behavior among residents. SEM effectively integrates factor analysis and path analysis, addressing the complex relationships between latent and observed variables [28]. It is widely utilized in the study of travel behavior [29,30]. The methodological framework comprises both the measurement model and the structural model.

The measurement model (Equation 1 and 2) is utilized to establish the scale relationship between latent variables and observed indicators.


$$Y=\wedge_{y}\eta +\varepsilon$$ (1)

$$X=\wedge_{x}\xi +\;\delta$$ (2)

Equation (1) represents the measurement equation for endogenous variables, while Equation (2) represents the measurement equation for exogenous variables.

The structural model (Equation 3) illustrates the causal relationships between equivalent latent variables, with the strength of the influence assessed using a standardized coefficient.


η=Bη+Γξ+ζ (3)

In Equation (3), η is a p × 1 matrix of endogenous observed variables; X is a q × 1 matrix of exogenous observed variables; B is the endogenous path coefficient matrix; Γ is an exogenous path coefficient matrix; ζ is the error matrix of endogenous observed variables.

**Fig 2:**
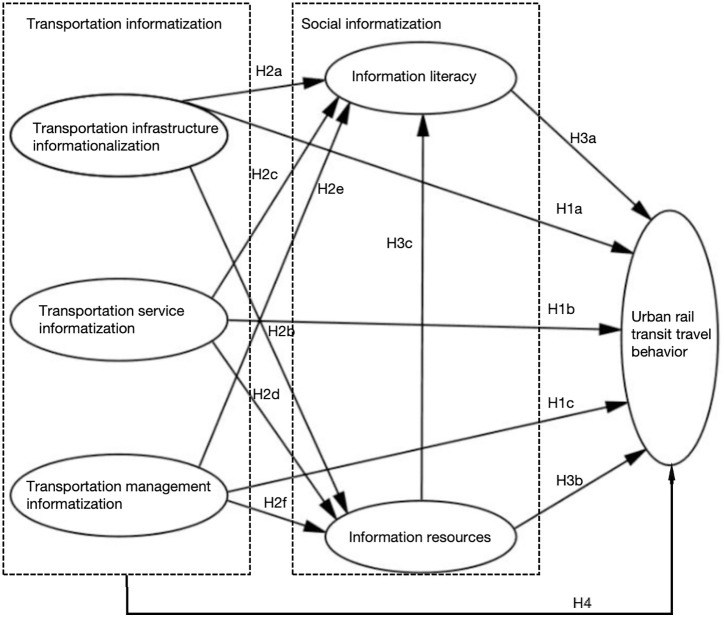
Conceptual model.

This study proposes hypotheses based on the existing theoretical framework and constructs a conceptual model using AMOS 25.0 (Fig 2), focusing on verifying the interaction mechanism between transportation informatization, social informatization, and residents’ urban rail transit travel behavior.

As mentioned above, the development of mobile internet provides a foundation for transportation informatization and influences residents’ urban rail transit travel behavior through the informatization of infrastructure, services, and management. Transportation infrastructure informatization is crucial to improving the travel convenience of residents’ urban rail transit [31], while transportation informatization serves as a bridge between infrastructure services and residents’ urban rail transit travel behavior [32], thereby improving the availability of information. The informatization of traffic management ensures the efficient operation of the system through effective control, coordination, and management. Meanwhile, transportation informatization fosters social informatization by improving information literacy and expanding information resources. It plays a critical role in control, coordination, promotion, and management [33]. Based on these analyses, the following hypotheses are proposed:

H1 Transportation informatization has a significant influence on urban rail transit travel behavior.

H1a The transportation infrastructure informatization has a direct and significant influence on urban rail transit travel behavior.

H1b The transportation service informatization has a direct and significant influence on urban rail transit travel behavior.

H1c The transportation management informatization has a direct and significant influence on urban rail transit travel behavior.

The second hypothesis pertains to the overall informatization of society as a consequence of the mobile internet. The level of development of transportation infrastructure informatization and transport service informatization is an important indicator of the level of information literacy of the population, and together they determine the ability of the residents to acquire, process, and apply information [34]. The level of traffic management informatization is not only affected by the information literacy of traffic service personnel, but also by the difficulty of implementation is closely related to the information literacy of travelers. In addition, transportation infrastructure informatization and traffic service informatization are the key conditions for the formation of mobile internet information resources, which to some extent affect the information availability and acceptability of information. Traffic management informatization is an effective means to develop, provide, control, reasonably release and distribute mobile internet information resources [35], which filters and controls the information generated by transportation infrastructure and transportation services to meet the diverse needs of different travelers [36]. Based on this analysis, the following hypotheses are proposed:

H2 There is a significant influence of transportation informatization on social informatization.

H2a The transportation infrastructure informatization has a direct and significant influence on the information literacy of urban residents.

H2b The transportation infrastructure informatization has a direct and significant influence on information resources.

H2c The transportation services informatization has a direct and significant influence on the information literacy of urban residents.

H2d The transportation services informatization has a direct and significant influence on the information resources.

H2e The transportation management informatization has a direct and significant influence on information literacy.

H2f The transportation management informatization has a direct and significant influence on information resources.

Existing studies indicate that age and education significantly influence residents’ informational travel behavior, suggesting that travelers adjust revise their travel strategies based on the information they gather throughout the travel process [37]. Age and education are critical factors affecting residents’ informational travel behavior, leading them to modify their travel strategies according to the information they have collected during the travel process [38]. Additionally, the abundance of transportation information advancements in transportation information technology also impact individuals’ travel behavior [39]. Based on these findings, the following hypotheses are proposed:

H3 Social informatization significantly influences residents’ urban rail transit travel behavior.

H3a Residents’ information literacy has a direct and significant impact on urban rail travel behavior.

H3b The development of information resources has a direct and significant impact on urban rail travel behavior.

H3c The construction of urban information resources has a direct and significant impact on residents’ information literacy.

The mobile internet plays a crucial role in shaping travel behavior by providing essential information resources [40]. These resources include access to accurate transportation information, travel times, and weather forecasts, all of which are facilitated by mobile internet and collectively influence residents’ travel decisions [41,42]. Based on this, the following hypotheses are proposed:

H4 Social informatization plays a crucial mediating role in the relationship between the development of transport informatization and residents’ urban rail transit travel behavior.

Based on the literature review, three potential variables have been identified: transportation informatization, social informatization, and residents’ urban rail transit travel behavior. Additionally, several explanatory factors have been recognized, including transportation infrastructure informatization, transportation service informatization, transportation management informatization, information literacy, information resources, pre-travel preparation, and in-travel adjustments. A measurement scale consisting of 17 items was developed. Table 2 summarizes all the variables utilized in the analysis. A Likert scale was employed, categorized into 5 levels: Strongly Disagree, Disagree, Neutral, Agree, and Strongly Agree, with corresponding values of 1, 2, 3, 4, and 5, respectively.

**Table 2:** Variables used in the analysis.

Variable	Description
TI Transportation informatization	
TII Transportation infrastructure informatization	TII1: Do you think that mobile internet has improved the efficiency of space utilization in urban rail transit stations?
	TII2: If you have an emergency at a public transportation station, can your smartphone help you get help or information faster?
	TII3: Do you think the coverage of the current information infrastructure facilities for urban rail transportation meets the needs of travel?
TSI Transportation service informatization	TSI1: When using mobile applications related to urban rail transport, how user-friendly is the interface?
	TSI2: Are you satisfied with the accuracy of the information provided by these application systems?
TMI Transportation management informatization	TMI1: Do you think that the information provided by the urban rail transit management department via mobile internet is helpful for your travel decisions?
	TMI2: Are you satisfied with the timeliness of the urban rail traffic management’s response to user feedback on the mobile internet?
SI Social informatization	
IL Information literacy	IL1: Are you able to effectively gather travel information using mobile internet tools?
	IL2: Do you frequently encounter problems when planning your travel using mobile internet?
IR Information resources	IR1: To what extent do you think that travel information resources obtained through mobile internet can assist you during your travels?
	IR2: How do you deal with information overload when using mobile internet to access travel information?
TB Travel Behavior Scale	
TP Pre-travel preparation	TP1: Do you actively consult digital maps, travel routes, and road conditions before your trip?
	TP2: What is your perspective on the impact of traffic information accessed via mobile internet in alleviating pre-travel anxiety?
TA Travel adjustments during the trip	TA1: When faced with unforeseen travel changes, how efficient is it for you to replan your itinerary using mobile internet?
	TA2: How difficult do you find it to change your travel plans using mobile internet tools?
	TA3: Do you often use mobile navigation systems while traveling?
	TA4: Do you often rely on smartphones for communication while traveling?

### 3.4. Modeling process

The reliability and validity of the data were assessed using SPSS 25.0. The results indicated a high Cronbach’s alpha of 0.727 to 0.900, confirming the reliability of the questionnaire. The Kaiser-Meyer-Olkin test (0.881) and Bartlett’s test (p = 0.000) showed good correlation among the 17 variables. Subsequently, factor analysis was conducted on the latent variables. The composite reliability (CR) for each of the three latent variables (transportation informatization, social informatization, and residents’ urban rail transit travel behavior) was in the range of 0.723–0.870, and the average variance extracted (AVE) values were in the range for 0.57–0.64, indicating excellent composite reliability and convergent validity for the combination of latent variables (Table 3).

**Table 3:** Factor Loadings, Reliability, and Validity of the Formal Scale.

Variable	Items	Standardized Factor Loadings	Correlation of Measurement Items with Variables	CR	AVE	Cronbach’s α
Transportation infrastructure informatization	TII1	0.768	0.615	0.7882	0.5542	0.764
	TII2	0.767	0.631			
	TII3	0.696	0.601			
Transportation service informatization	TSI1	0.766	0.632	0.7274	0.5716	
	TSI2	0.746	0.646			
Transportation management informatization	TMI1	0.742	0.663	0.7281	0.5725	
	TMI2	0.771	0.676			
Information literacy	IL1	0.776	0.745	0.7565	0.6084	0.726
	IL2	0.784	0.742			
Information resources	IR1	0.818	0.737	0.7537	0.6054	
	IR2	0.736	0.742			
Pre-travel preparation	TP1	0.78	0.821	0.7801	0.6396	0.898
	TP2	0.819	0.828			
Travel adjustments during the trip	TA1	0.822	0.783	0.8717	0.6294	
	TA2	0.861	0.815			
	TA3	0.81	0.786			
	TA4	0.834	0.789			

Due to the complexity of the interactions among the variables, this study employed SEM, which is notable for its ability to treat a variable as both an outcome and an explanatory variable simultaneously. SEM also effectively identifies mediating effects by distinguishing between direct, indirect, and aggregate effects. This research leverages the unique advantages of structural equation modeling and synthesizes insights from previous studies to investigate the factors influencing residents’ urban rail transit travel behavior and its relationship with mobile internet usage. Based on the analysis results of the initial model, the Bollen-Stine bootstrap method was applied to refine the model [43], achieving a fit that meets rigorous scientific criteria, as evidenced by the indices presented in Table 4. Previous studies typically indicate an adequate model fit by CFI values above 0.90 and RMSEA values below 0.08 [44]. All other indicators also met the necessary requirements [45–47]. The model’s goodness of fit ensures the robustness and credibility of our research findings.

**Table 4:** Model’s goodness of fit.

Model’s goodness of fit	x^2^/df	RESEA	NFI	TLI	CFI	GFI	AGFI	IFI
Original value	4.066	0.069	0.927	0.917	0.943	0.948	0.911	0.944
Corrected value by Bollen-Stine Ideal value Fitting evaluation	2.443	0.047	0.957	0.961	0.974	0.973	0.952	0.974
Ideal value	<3.00	<0.08	>.90	>.90	>.90	>.90	>.90	>.90
Fitting evaluation	ideal	ideal	ideal	ideal	ideal	ideal	ideal	ideal

## 4. Results

### 4.1. Model results

**Fig 3:**
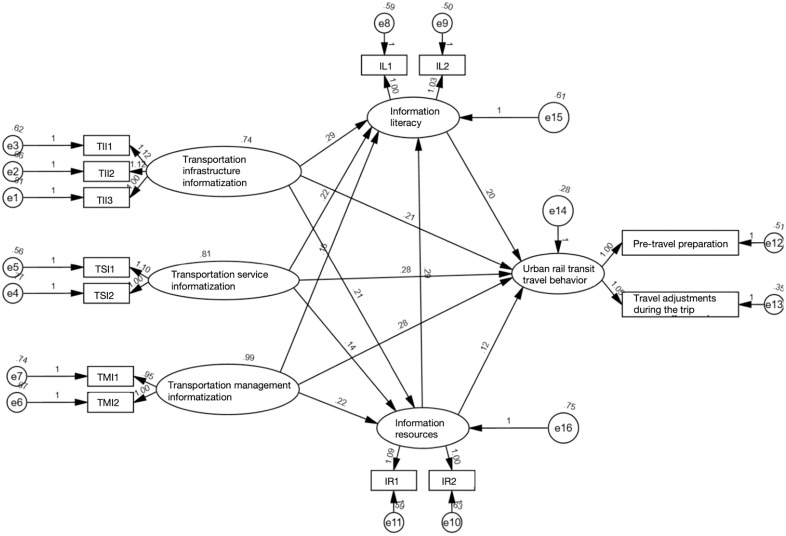
The results of SEM.

**Fig 4:**
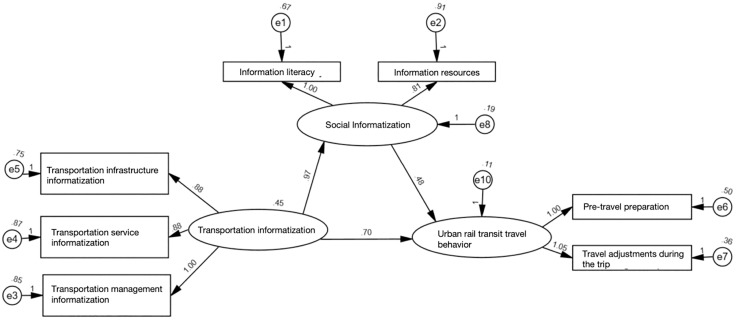
Relational graph of aggregate variables.

The final structural equation model is illustrated in Fig 3. Table 5 presents the direct, indirect, and total effects of each variable pair within the model, confirming that all 12 sub-hypotheses of H1, H2, and H3 are supported. Furthermore, the mediating effects of social informatization were analyzed using the methodology proposed by Baron, with Fig 3 serving as the baseline model [48]. As shown in Table 6, after incorporating social informatization, transportation informatization remains significantly related to residents’ urban rail travel behavior; however, this relationship is notably weakened, with the path coefficient value decreasing from 0.95 to 0.54. Therefore, social informatization plays a partial mediating role between the two, supporting hypothesis H4. Fig 4 illustrates the interrelationship among transportation informatization, social informatization, and urban rail travel behavior. The figure indicates that the direct effect of transportation informatization on urban rail transit travel behavior is 0.54, the indirect effect is calculated as 0.83*0.43 = 0.36, and the total effect is 0.90.

**Table 5:** Standardized total effects, direct effects, and indirect effects of the SEM.

Variables	Direct	Indirect	Total
Transportation Management Informatization → Information Resources	0.228	—	0.228
Transportation Management Informatization → Information Literacy	0.148	0.065	0.213
Transportation Management Informatization → Urban Rail Transit Travel Behavior	0.339	0.082	0.421
Transportation Service Informatization → Information Resources	0.133	—	0.133
Transportation Service Informatization → Information Literacy	0.21	0.038	0.248
Transportation Service Informatization → Urban Rail Transit Travel Behavior	0.311	0.077	0.388
Transportation Infrastructure Informatization → Information Resources	0.17	—	0.170
Transportation Infrastructure Informatization → Information Literacy	0.252	0.048	0.300
Transportation Infrastructure Informatization → Urban Rail Transit Travel Behavior	0.18	0.094	0.274
Information Resources → Information Literacy	0.284	—	0.284
Information Resources → Urban Rail Transit Travel Behavior	0.144	—	0.144
Information Literacy → Urban Rail Transit Travel behavior	0.232	0.066	0.298

**Table 6:** Checkout Procedure of Intervening Variable.

Steps	Relationships between variables	Path coefficients and significance
step1	Transportation Informatization → Social Informatization	0.902***
step2	Transportation Informatization → Urban Rail Transit Travel Behavior	0.950***
step3	Social Informatization → Urban Rail Transit Travel Behavior	0.955***
step4	Transportation Informatization → Social Informatization	0.827***
	Transportation Informatization → Urban Rail Transit Travel Behavior	0.541***
	Social Informatization → Urban Rail Transit Travel Behavior	0.427**

### 4.2. Discussion on results

The data presented in Table 6 indicate that transportation informatization has a significant overall impact (0.95) on urban rail transit travel behavior, with distinct mechanisms operating across three dimensions: transportation infrastructure informatization, transportation service informatization, and transportation management informatization. First, transportation infrastructure informatization directly affects urban rail transit travel behavior (0.18). This finding complements the study by Huang et al (2024) on the impact of mobile applications on travel route choice, collectively revealing the multilevel effects of informatization technology interventions on urban rail transit travel behavior [49]. In contrast to existing studies that focus on technology application scenarios in small and medium-sized cities [50], this study demonstrates that high-density technology penetration in megacities significantly enhances the shaping effect of transportation infrastructure informatization on urban rail transit travel behavior. Secondly, within the service dimension, service informatization enriches the passenger experience through real-time updates on train schedules and route planning. This mechanism aligns theoretically with the service response model proposed by Shen et al. (2020) [51]. The study further reveals the indirect reinforcing effect of service informatization on information literacy, indicating that, under the high penetration rate of mobile internet technology, smart terminals may serve as persistent vehicles for cultivating residents’ information competencies. This finding expands the cognitive boundaries of existing research regarding the functional attributes of service informatization. Third, in the management dimension, traffic management informatization demonstrates a significant overall impact on urban rail transit travel behavior (0.955). Through data-driven prediction, guidance, control, and coordination systems, it optimizes the entire travel process. Its minute-level response mechanism plays a crucial role in alleviating congestion and enhancing travel flexibility [52]. This aligns with the methodology of Wang et al. (2024), who emphasized the significance and practical application of transportation management informatization, highlighting the necessity of effectively utilizing information technology to promote system optimization and sustainable development in traffic management [53]. Moreover, this impact directly influences urban rail transit travel behavior through the key dimensions of information literacy and information resources. This mechanism not only provides a quantitative basis for assessing traffic management effectiveness but is also essential for developing smart city strategies.

The study also confirmed that transportation informatization has a positive combined effect on social informatization, with an impact value of 0.902. Its pathways of influence exhibit multidimensional characteristics. Specifically, the informatization of transportation infrastructure significantly enhances the information literacy of travelers, thereby supporting hypothesis H2a. This finding cross-validates Van Wee’s (2013) theoretical framework, which posits that transportation infrastructure informatization enhances the literacy of the public’s ability to acquire, process, and apply information related to transportation and mobility [54]. In contrast, this study further validates the practical implications of this effect in Chinese megacities, highlighting its universality. Meanwhile, the informatization of transportation services significantly impacts the enhancement of information literacy and access to information resources, thereby supporting hypotheses H2c and H2d. This finding aligns with the conclusions of most existing studies, which emphasize that the popularity and convenience of smartphones, computers, and Wi-Fi play a crucial role in shaping residents’ information literacy and their access to information resources. Contrasting with Lam et al. (2013) regarding information accessibility [55], this study underscores the unique contribution of travel informatization to the dynamic optimization of the travel experience. Specifically, it highlights the direct impact of real-time information updates on travel decision support, thereby enriching the analytical perspective at the service level. Furthermore, the informatization of traffic management significantly influences social informatization, which is evident not only in the improvement of information literacy among traffic personnel, but also in the challenges of implementation and resource integration complexity. As the central force controlling information resources, traffic management informatization optimally enhances urban rail transit travel behavior through the development, utilization, transmission, dissemination, and effective coordination of technology.

Social informatization enhances residents’ information literacy, enabling individuals to better understand and engage with integrated transportation services, thereby optimizing their travel decisions [56]. By making substantial investments in information resource infrastructure, developed countries have successfully reduced traffic congestion, lowered traffic planning and management costs, and achieved seamless integration of various modes and systems of transport. This study further confirms that both the quality and quantity of information resources significantly impact residents’ travel efficiency, highlighting the crucial role of mobile internet in modern transportation systems.

## 5. Conclusions

Urban rail transit travel behavior in Chinese megacities is experiencing significant transformations, driven by technological advancements. This study systematically investigates the impact of mobile internet on urban rail transit travel behavior among Chongqing residents through a comprehensive literature review, questionnaire surveys, and SEM analysis. The findings reveal two key insights. First, transportation informatization significantly shapes urban rail transit travel behavior. SEM analysis indicates that the total effect of transportation informatization on urban rail transit travel behavior is 0.90, comprising a direct effect of 0.54 and an indirect effect of 0.36. Specifically, transportation infrastructure informatization directly influences urban rail transit travel behavior with a direct effect of 0.18, while transportation management informatization optimizes the travel process through data-driven predictions, yielding a total effect of 0.421. Additionally, transportation service informatization indirectly shapes urban rail transit travel behavior by improving information literacy and access to information resources, with an indirect effect of 0.077. Second, social informatization acts as a mediator in the relationship between transportation informatization and urban rail transit travel behavior. The SEM analysis shows that the interrelationships among transportation informatization (TI), social informatization (SI), and urban rail transit travel behavior (TB) yield path coefficients of 0.827 (TI → SI), 0.427 (SI → TB), and 0.54 (TI → TB after incorporating SI). Upon introducing social informatization, the effect of transportation informatization on urban rail transit travel behavior remains statistically significant but weakens considerably, with the path coefficient decreasing from 0.95 to 0.54. This indicates that social informatization mediates the relationship by enhancing information resources (with a total effect of 0.210) and improving information literacy (with a total effect of 0.232). This mediating pathway highlights the pivotal role of social informatization in linking technology and behavior. These results not only enhance the theoretical understanding of the impact of mobile internet on urban rail transit travel behavior but also provide new insights for urban planning and traffic management. They emphasize the importance of incorporating social informatization into the design of transportation systems and highlight the necessity of improving information literacy in policy formulation.

Based on the findings of the study, we recommend that policymakers prioritize the implementation of mobile internet technology within the urban transportation sector. This strategy aims to enhance transportation informatization and improve residents’ experiences with rail travel. Additionally, we suggest strengthening information literacy education to bridge the information gap, ensuring that residents can fully benefit from the conveniences offered by mobile internet. Furthermore, future research should investigate the impact of changes in residents’ urban rail transit travel behavior on urban spaces, thereby supporting the sustainable development of urban environments in the digital economy era.

With the continuous evolution of mobile internet technology, observing and analyzing the long-term changes in residents’ urban rail transit travel behavior patterns has become necessary, providing a solid foundation for the renewal and optimization of urban spaces. This study explored SEM to examine the influence of mobile internet on urban rail transit travel behavior and the mediating effect of social informatization. However, a single model has limitations in revealing the dynamic changes in the mechanisms of action or in making causal inferences. Future research should integrate innovative methods, such as machine learning, to explore the internal mechanisms affecting residents’ urban rail transit travel behavior thereby enriching related research findings. In addition, simple sampling methods do not adequately account for individual heterogeneity, and traditional questionnaire techniques are limited by response time and emotional context. Therefore, future research will incorporate wearable devices to enhance data accuracy and reduce survey errors caused by subjective preferences, while also exploring the impact pathways using dynamic learning mechanisms and examine the long-term effects of changes in urban rail transit travel behavior on urban spatial organization, thus providing more comprehensive support for sustainable urban development in the information age.

## Supporting information

S1 File Annexure. (DOCX)
