# Supplementary material for: Analyzing the influence of mobile internet on urban rail transit travel behavior in Chongqing, China: Social informatization as mediating variables
Source: PLoS One. 2025 Nov 20;20(11):e0331977. doi: 10.1371/journal.pone.0331977 (PMC12633907; doi:10.1371/journal.pone.0331977)
Supplement: S1 File — (DOCX) [file pone.0331977.s001.docx]

**Annexure**

Questionnaire on urban rail transit travel behavior under the influence of mobile internet

Dear Sir/Madam,

I am a student from Chongqing University. In order to have a deeper understanding of the impact of mobile internet on travelers’ demand for and satisfaction with rail transit stations, I conducted this survey. Thank you for your help during your busy schedule. The information you provide will only be used for statistical analysis and scientific research, absolutely guarantee the privacy and security of the data you fill in, sincerely thank you for your cooperation!

Table 7 instructions: Please indicate your actual situation by marking “✓” in the appropriate boxes.

Part A. Personal Information Section

Table 7. Questionnaire on Socio-demographic Characteristics.

| 1. Your gender | □Male | □Female |  |  |  |
| --- | --- | --- | --- | --- | --- |
| 2. Your highest education | □Junior high school or less | □High school or Technical secondary school | □Junior college | □Undergraduate | □Graduate or higher |
| 3. Your age | □19-30 | □31-50 | □Over 50 |  |  |
| 4. Your years of work experience (years) | □1-2 | □3-5 | □6-9 | □over 10 |  |
| 5. The type of work you are engaged in | □School student | □Commercial trade | □Government agencies and public institutions | □Other |  |
| 6. Your years of mobile internet use (year） | □1-2 | □3-5 | □6-9 | □Over 10 |  |
| 7. How often do you take public transport | Daily | Multiple times a week | Once a week | Multiple times a month |  |

Part B. Questionnaire Section

Table 8 instructions: This table includes variables (with sub-dimensions) and a 5-point Likert scale (1=Strongly Disagree, 2=Disagree, 3=Neutral, 4=Agree, 5=Strongly Agree). Rate each question by selecting the corresponding number. For the multi-select and open-ended questions, answer based on your actual situation.

Table 8. Variable Definitions and Measurement Scale.

| Serial Number | Question Items | Strongly Disagree | Disagree | Neutral | Agree | Agree Strongly |
| --- | --- | --- | --- | --- | --- | --- |
| TI Transportation informatization | | | | | | |
| TII  Transportation infrastructure informatization | TII1 Do you think that mobile internet has improved the efficiency of space utilization in urban rail transit stations? | 1 | 2 | 3 | 4 | 5 |
|  | TII2: If you have an emergency at a public transportation station, can your smartphone help you get help or information faster? | 1 | 2 | 3 | 4 | 5 |
|  | TII3: Do you think the coverage of the current information infrastructure facilities for urban rail transportation meets the needs of travel? | 1 | 2 | 3 | 4 | 5 |
| TSI  Transportation service informatization | TSI1: When using mobile applications related to urban rail transport, how user-friendly is the interface? | 1 | 2 | 3 | 4 | 5 |
|  | TSI2: Are you satisfied with the accuracy of the information provided by these application systems? | 1 | 2 | 3 | 4 | 5 |
| Serial Number | Question Items | Strongly Disagree | Disagree | Neutral | Agree | Agree Strongly |
| TMI  Transportation management informatization | TMI1: Do you think that the information provided by the urban rail transit management department via mobile internet is helpful for your travel decisions? | 1 | 2 | 3 | 4 | 5 |
|  | TMI2: Are you satisfied with the timeliness of the urban rail traffic management’s response to user feedback on the mobile internet? | 1 | 2 | 3 | 4 | 5 |
| SI Social informatization | | | | | | |
| IL  Information literacy | IL1: Are you able to effectively gather travel information using mobile internet tools? | 1 | 2 | 3 | 4 | 5 |
|  | IL2: Do you frequently encounter problems when planning your travel using mobile internet? | 1 | 2 | 3 | 4 | 5 |
| IR  Information resources | IR1: To what extent do you think that travel information resources obtained through mobile internet can assist you during your travels? | 1 | 2 | 3 | 4 | 5 |
|  | IR2: How do you deal with information overload when using mobile internet to access travel information? | 1 | 2 | 3 | 4 | 5 |
| TB Travel Behavior Scale | | | | | | |
| TP  Pre-travel preparation | TP1: Do you actively consult digital maps, travel routes and road conditions before your trip? | 1 | 2 | 3 | 4 | 5 |
|  | TP2: What is your perspective on the impact of traffic information accessed via mobile internet in alleviating pre-travel anxiety? | 1 | 2 | 3 | 4 | 5 |
| TA  Travel adjustments during the trip | TA1: When faced with unforeseen travel changes, how efficient is it for you to replan your itinerary using mobile internet? | 1 | 2 | 3 | 4 | 5 |
|  | TA2: How difficult do you find it to change your travel plans using mobile internet tools? | 1 | 2 | 3 | 4 | 5 |
|  | TA3: Do you often use mobile navigation systems while traveling? | 1 | 2 | 3 | 4 | 5 |
|  | TA4: Do you often rely on smartphones for communication while traveling? | 1 | 2 | 3 | 4 | 5 |
| Which aspects of rail transit stations do you think need most improvement under mobile internet influence? (Multi-select): | | | | | | |
| □Environmental comfort □Spatial practicality □Facility practicality □Service quality  □Functional diversity □Age-friendly design □Smart experience □Safety □Other | | | | | | |
| What negative issues do you think currently exist in urban rail transit station areas? | | | | | | |

Thank you very much for taking the time to fill out this questionnaire! Your cooperation is greatly appreciated!
